# Diatom-based treatment of geothermal brines to selectively remove silicon, retain lithium for downstream recovery, and produce omega-3 fatty acids

**DOI:** 10.1016/j.isci.2026.117965

**Published:** 2026-10-26

**Authors:** Mohammed Rehmanji, Alastair Skeffington, Karen A. Hudson-Edwards, Laura Newsome

**Affiliations:** 1 Camborne School of Mines & Environment and Sustainability Institute, University of Exeter, Penryn, Cornwall TR10 9FE, UK; 2 Department of Biological and Environmental Sciences, University of Stirling, Stirling, Scotland

**Keywords:** lithium, geothermal brine, diatoms, Si uptake, omega-3 fatty acids, circular bioeconomy

## Abstract

Silica (SiO2) scaling in geothermal brines processing can hinder energy production and downstream lithium (Li) recovery. Here, we investigate diatom-based selective silicon (Si) removal from brines to support downstream recovery of lithium (Li) and generating biomass for valorization. Screening of six diatom strains identified *Halamphora* sp. as the most effective for Si removal. When cultivated in geothermal brine supplemented with urea, *Halamphora* sp. removed 34 mg L^−1^ of Si (97.14%) within 48 h, while retaining over 99% of Li (75 mg L^−1^) in solution. Urea supplementation also enhanced biomass production, reaching 0.45 g L^−1^. The resulting biomass contained the omega-3 fatty acids eicosapentaenoic acid (EPA; 47 mg g^−1^) and docosahexaenoic acid (DHA; 24 mg g^−1^). These results demonstrate the potential for coupling biological Si removal and Li retention with production of value-added biomass during geothermal brine processing.

## Highlights

• *Halamphora* sp. selectively removes silicon from Cornish geothermal brine
• Semi-continuous urea feeding achieves 97% silicon removal within 48 h
• Bioprocess retains >99% lithium in solution for downstream recovery
• Urea-fed biomass yields high-value omega-3 fatty acids (EPA and DHA)

## Graphical abstract

**Figure:**
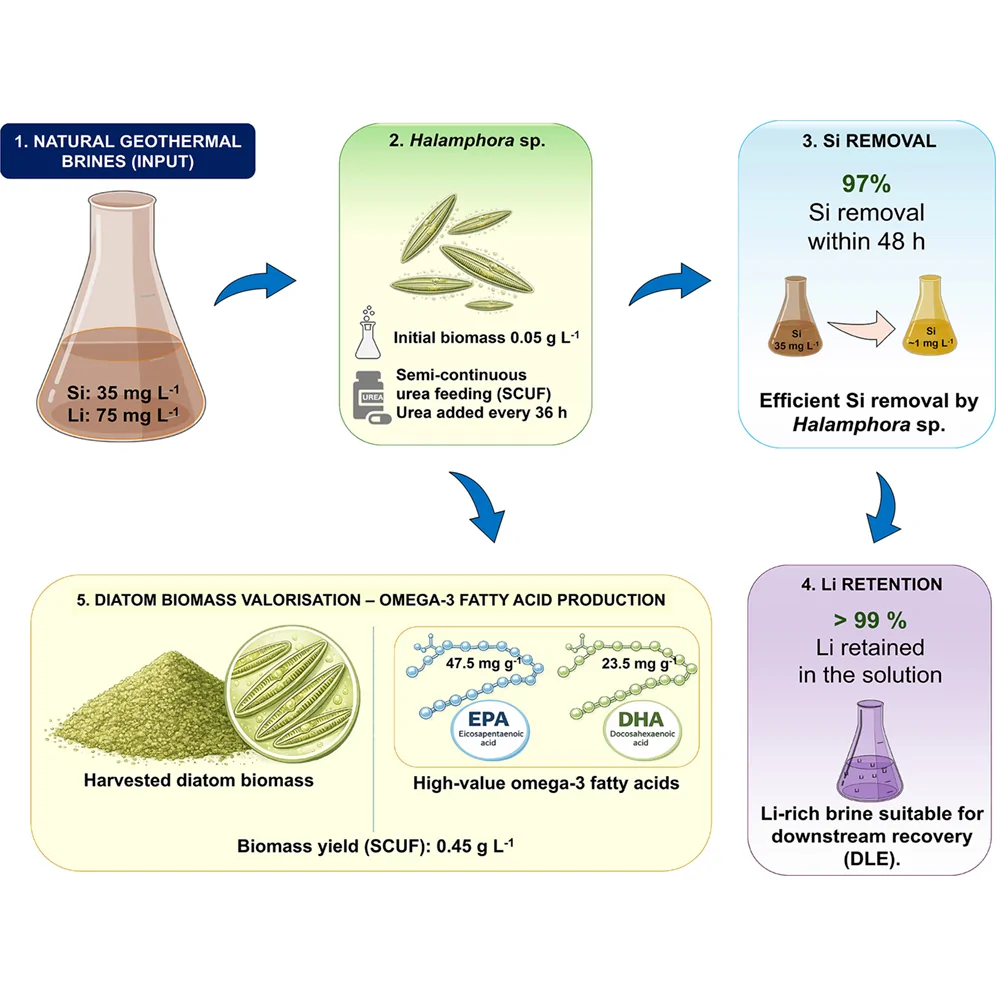

## Teaser

Chemical engineering; Geochemistry; Environmental biotechnology; Biotechnology

## Introduction

Diatoms are among the most abundant and productive photosynthetic microorganisms on Earth, playing a central role in the conversion of light energy and carbon dioxide into sustainable bioresources.^1^ A defining characteristic of diatoms is their requirement for dissolved silicon (Si), which they assimilate as silicic acid (Si (OH)_4_) to construct their intricately patterned cell walls, or frustules. Through this process, diatoms contribute substantially to the global biogeochemical cycling of Si.^2^ The cellular mechanism of Si handling in diatoms involves a tightly regulated, multi-stage biomineralization pathway. At low ambient concentrations (<30 μM), silicic acid uptake is mediated by energy-dependent, sodium-coupled silicon transporters (SITs), driven by a Na^+^ electrochemical gradient maintained by cellular respiration or photosynthesis.^3^ At hyper-saturating concentrations such as those encountered in mineralized waters uptake shifts predominantly toward concentration-dependent passive diffusion and fluid-phase endocytosis.^4^ Following uptake, silicic acid is temporarily stored in intracellular soluble pools before being transported into specialized, membrane-bound organelles termed silica deposition vesicles (SDVs). Within the acidic environment of the SDV, specialized organic matrix proteins and macromolecules—specifically silaffins, long-chain polyamines (LCPAs), and silacidins—catalyze and template the rapid polycondensation of monomeric silicic acid into amorphous silica (SiO_2_·*n*H_2_O) frustules.

Varying concentrations of environmental Si exert a profound influence on diatom physiology, cell cycle progression, and overall metabolic flux. Under Si limitation or starvation, diatoms experience cell-cycle arrest at the G1/S and G2/M transition checkpoints, suppressing cell division, reducing cell wall thickness, and inducing a metabolic re-routing of carbon toward triacylglycerol (TAG) and lipid synthesis as stress-response energy reserves. Conversely, when exposed to high or excess Si concentrations, such as those characteristics of geothermal brines, diatoms maintain uninhibited cell division, exhibit elevated biosilicification rates, and construct denser, highly mineralized frustules without undergoing cell-cycle arrest, provided ionic strength and osmotic stress remain within physiological tolerance limits. This physiological plasticity and high capacity for Si biomineralization make diatoms ideal biological agents for targeted Si capture in industrial fluids.

High concentrations of dissolved Si (typically 1.5–150 mg L^−1^) are a persistent challenge in geothermal systems, where Si precipitation and scaling significantly reduce heat-transfer efficiency, obstruct pipelines, and increase operational and maintenance costs.^5^ Geothermal brines are high-temperature, mineral-rich fluids extracted primarily for renewable energy production, but they have recently gained attention as potential sources of critical raw materials, particularly lithium (Li).^6^ Lithium is listed as a critical metal in several national criticality assessments, including those of the UK, US, Canada, and Australia, due to its essential role in lithium-ion batteries for electric vehicles and grid-scale energy storage.^7^^,^^8^^,^^9^ Securing projected global demand will require an approximately 8-fold increase in lithium production by 2040.^10^ The state of art technology to extract Li from brine is direct Li extraction (DLE). DLE integrates various thermal and electrochemical processes, utilizing ion-exchange resins or sorption-based methods to extract Li directly from brines.^11^ High Si concentrations have been shown to significantly reduce Li recovery efficiency in brines; for example, raising Si levels from 0 to 150 mg L^−1^ resulted in a decline in Li recovery from 6.8% to 4.5%.^5^ More recent electrochemical studies demonstrate that Si deposition causes impurity-induced scaling, reduces active surface area, impedes Li^+^ intercalation kinetics, and degrades electrode crystal structures, collectively impairing extraction performance.^12^ Effective Si removal is therefore critical not only for geothermal energy production but also for enabling efficient Li recovery from geothermal resources.

Conventional Si mitigation strategies, such as chemical precipitation and acidification, are often inefficient and generate large volumes of waste.^13^ Sorption-based approaches have also been investigated; however, challenges associated with sorbent regeneration and Si desorption have limited their industrial applicability.^14^ Previous bioprocessing strategies for geothermal brines have focused primarily on mitigating divalent cation scaling (Ca^2+^ and Mg^2+^) using bacterial microbially induced carbonate precipitation (MICP). While MICP effectively precipitates metal carbonates, it does not remove dissolved Si, which is an equally severe, yet chemically distinct, challenge to DLE infrastructure. In this context, the use of diatoms as a biological Si-removal strategy represents a potentially sustainable alternative. By naturally incorporating dissolved Si into their frustules, diatoms offer a low-waste, biologically driven approach to mitigating Si scaling in geothermal brines, an application that remains largely unexplored.

In addition to Si removal, diatom cultivation offers potential co-benefits, marine diatoms have been widely studied for applications including metal removal,^15^ nanoparticle synthesis,^16^ and as feedstocks for renewable fuels and high-value biorenewables.^17^^,^^18^^,^^19^ Notably, diatoms can produce commercially valuable long-chain polyunsaturated fatty acids (LC-PUFAs), such as eicosapentaenoic acid (EPA) and docosahexaenoic acid (DHA), which are widely used in pharmaceutical and nutraceutical industries. These ω-3 fatty acids typically account for 3%–5% of diatom dry cell weight (DCW), with reported productivities of approximately 56 mg L^−1^ day^−1^ in model species, highlighting their potential as sustainable, high-value co-products.^18^

In the present study, we evaluated the potential of *Halamphora* sp., alongside other diatom species, for the bioprocessing of geothermal brines. Specifically, we investigated their capacity to selectively remove dissolved Si as a strategy to mitigate scaling, while simultaneously assessing their ability to support lithium retention for downstream recovery and generate valuable ω-3 fatty acids as co-products. The proposed bioprocess is designed for implementation downstream of heat exchange and upstream of DLE, providing a targeted pre-treatment step within the geothermal processing workflow.

## Results and discussion

### Effect of diatom treatment for Si removal from natural brine

We hypothesized that diatoms would remove Si from natural brine (NB), while other elements such as Li would remain in solution. Brine compositions are shown in Table 1. A Li-rich brine (75 mg L^−1^) was selected for use in these experiments, with a Si content of 35 mg L^−1^, and pH 7.2 (Table 1). Biomass production varied considerably among the six diatom species cultivated in NB (Figure 1A). *F. pelliculosa* showed the highest biomass accumulation, i.e., 2 g DCW L^−1^ (*p* < 0.01) over the cultivation period of 10 days, indicating good tolerance and adaptation to the saline brine environment. *Halamphora* sp., *C. weissflogii*, and *T. pseudonana* also achieved moderate biomass levels, whereas the two local isolates (*Swanpool isolate* and *Kennall Vale isolate*) displayed relatively low biomass production (*p* < 0.001 relative to top producers).

**Table 1:** Chemical composition (mg L^−1^) of natural brine used in this study

Analyte	Natural brine
pH	7.2 ± 0.5
EC (mS)	20 ± 1.5
Silicon (mg L^−1^)	35 ± 2.0
Calcium (mg L^−1^)	1,510 ± 2.4
Magnesium (mg L^−1^)	27 ± 1.3
Potassium (mg L^−1^)	205 ± 3.3
Sodium (mg L^−1^)	2,350 ± 3.2
Lithium (mg L^−1^)	75 ± 1.8
Boron (mg L^−1^)	13 ± 1.3
Copper (mg L^−1^)	2.0 ± 0.5
Iron (mg L^−1^)	2.5 ± 0.8
Manganese (mg L^−1^)	3.0 ± 0.4
Strontium (mg L^−1^)	29 ± 0.8
Nickel (mg L^−1^)	2.5 ± 0.9
Chloride (mg L^−1^)	7,710 ± 4.5
Sulfate (mg L^−1^)	270 ± 5.5
Phosphate (mg L^−1^)	142 ± 8.7

Data are represented as mean ± SD (standard deviation), *n* = 3 biological replicates.

**Figure 1:**
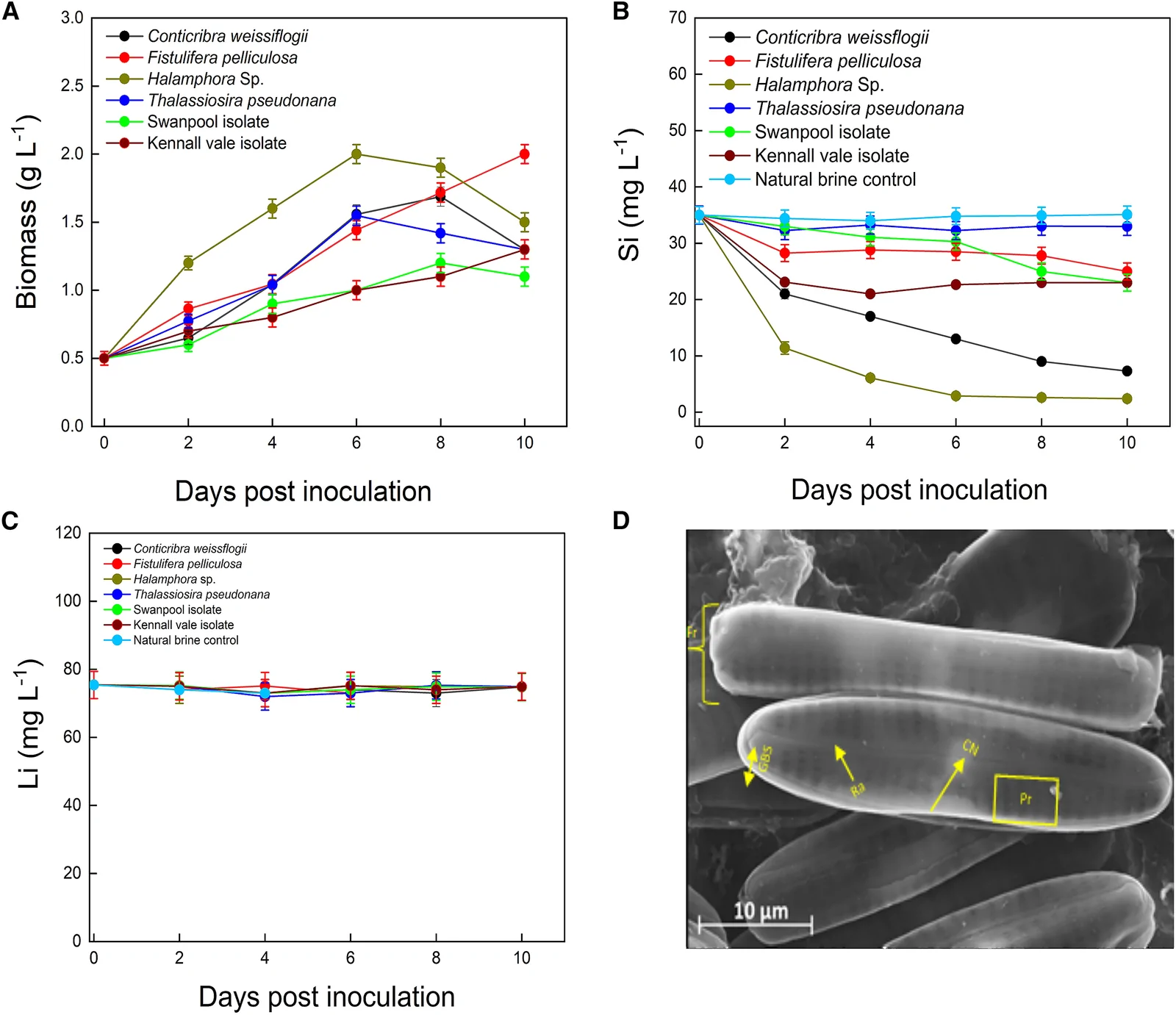
Biomass, Si, and lithium content in natural brine inoculated with *C. weissflogii* (black circles), *F. pelliculosa* (red circles), *Halamphora* sp. (olive green circles), *T. pseudonana* (blue circles), Swanpool isolate (green circles), Kennall vale isolate (dark red circles), and natural brine control without any diatoms (turquoise blue circles) Plots show concentration of biomass (A), Si (B), Li (C), and Intact *Halamphora* sp. cells in natural brine (Gbs, girdle bands; Pr, porous area; Ra, raphe; and Cn, central nodule) (D); images were acquired using an Everhart-Thornley detector (ETD) on the scanning electron microscope (SEM) operating, at an accelerating voltage of 25 kV, working distance of approximately 13 mm, magnification of 10^4^×, and a spot size of 5.0 nm. Data are represented as mean ± SD (standard deviation), *n* = 3 biological replicates. Student’s independent two-sample *t* tests (*df* = 4, two-tailed) were performed on the samples (*n* = 3 per condition) comparing treatment groups against the control. Significant values were determined on the basis of *p* < 0.0001.

The Si concentration in NB decreased substantially in the presence of *Conticribra weissflogii* and *Halamphora* sp. (Figure 1B) (*p* < 0.001). After 10 days, the Si removal efficiencies of each diatom strain were as follows: *Conticribra weissflogii* 81%, *Fistulifera pelliculosa* 29%, *Halamphora* sp. 93%, *Thalassiosira pseudonana* 6%, Swanpool isolate 34%, and Kennall Vale isolate 32%. Lithium remained in solution throughout the 10-day period in all experiments (Figure 1C). Although the experiments were conducted in glass flasks, control experiments (brine without diatoms) showed no significant change in Si concentration over the incubation period (Figure 1B), further a comparative analysis between untreated, filter sterilized, and autoclave brines also demonstrated minimal effect of abiotic Si precipitation (Figure S2), indicating negligible contribution from glass leaching and abiotic factors. Thus, the observed Si depletion is attributed primarily to diatom uptake.

The species-specific variation in biomass demonstrates that the diatoms were not equally suited to growth in natural geothermal brine. Si is a critical nutrient for diatoms, particularly during cell division.^21^ Although *Halamphora* sp. showed a moderate increase in biomass in NB, it nevertheless demonstrated the highest Si uptake among all species tested. *Halamphora* sp. exhibited higher Si over Li selectivity, achieving a separation factor (*SF*_Si/Li_) of >1,000 compared to 473 for *C. weissflogii* and 9 for *T. pseudonana* (Table S5). SEM images showed that *Halamphora* sp. cultured in NB retained an overall intact diatom morphology comparable to that observed in f/2 medium, with no obvious gross frustule deformation or dissolution (Figure 1D). This visual observation is consistent with the ability of *Halamphora* sp. to tolerate the brine environment; however, SEM alone does not directly demonstrate internal homeostasis or energy metabolism. These observations are consistent with those in previous reports demonstrating that *Halamaphora subtropica* and *Halamphora* species maintain high Si assimilation efficiency under chemically variable and nutrient-limited conditions, resulting in effective frustule formation even when growth is constrained.^22^^,^^23^ Notably, *Halamphora* sp. has been reported to achieve Si removal efficiencies of up to 99% in high Si aquatic systems.^23^ Together with the cell-free brine controls, which showed no significant change in dissolved silicon, these results support the interpretation that silicon depletion in inoculated cultures was associated with the presence of *Halamphora* sp., while a complete silicon mass balance was beyond the scope of this study. Based on Si uptake performance, *Halamphora* sp. was therefore selected for further investigation to optimize biomass production and Si removal from natural geothermal brine.

### Effect of initial inoculum density of *Halamphora* sp. on Si removal from natural brine

The rate of Si uptake is a critical parameter for a diatom-based brine treatment system as part of a Li extraction process. To evaluate this, varying biomass concentrations of *Halamphora* sp. were inoculated into NB (Figure 2). The results revealed a positive correlation between inoculum density and Si removal. At a low inoculum concentration of 0.05 g DCW L^−1^, approximately 24% of Si was removed from solution over a 10-day period (*p* < 0.01), whereas at a higher inoculum concentration of 1 g DCW L^−1^, up to 97% Si removal was achieved (*p* < 0.01). Increasing the *Halamphora* sp. inoculum density demonstrated a stepwise enhancement in biological selectivity, with the calculated (*SF*_Si/Li_) escalating from 0 up to >1,000 (Table S5). It is clear from these results that further diatom growth after inoculation in the brine (enhancing biomass beyond the initial inoculum) would be beneficial for enhancing Si uptake. Given that our objective was to maximize the Si uptake process while minimizing the initial investment in biomass, thereby conserving media and economic resources, we chose a resource-efficient initial inoculum density of 0.05 g DCW L^−1^ for all subsequent optimizations.

**Figure 2:**
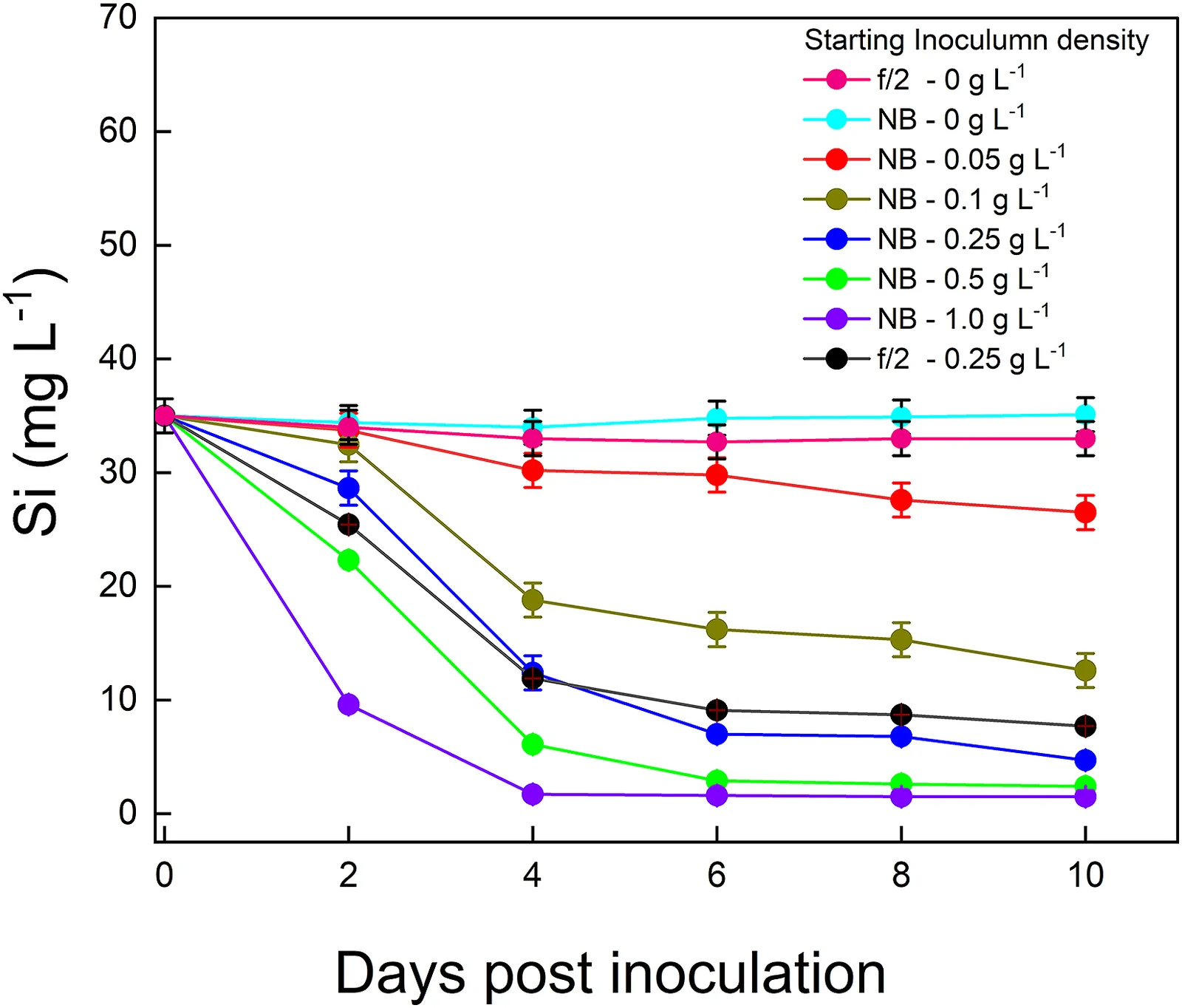
Si content in natural brine inoculated with *Halamphora* sp. with starting inoculum density 0 g DCW L^−1^ (cyan circles), 0.05 g DCW L^−1^ (red circles), 0.1 g DCW L^−1^ (olive green circles), 0.25 g DCW L^−1^ (blue circles), 5.0 g DCW L^−1^ (green circles), 1.0 g DCW L^−1^ (purple circles), and f/2 medium inoculated with 0.25 g DCW L^−1^ (black circle∗) Data are represented as mean ± SD (standard deviation), *n* = 3 biological replicates. Student’s independent two-sample *t* tests (*df* = 4, two-tailed) were performed on the samples (*n* = 3 per condition) comparing treatment groups against the control. Significant values were determined on the basis of *p* < 0.0001.

### Effect of urea supplementation on biomass

We noted that the NB contained relative high concentrations of P (Table 1), raising the possibility that growth in the NB might be N limited. Previous research identified urea as an appropriate N source for diatoms^18^ so its application was investigated using two approaches. Firstly, 26 mg L^−1^ (441 μM) of urea was added at the point of inoculation. Secondly, semi-continuous urea feeding (SCUF) was trialled, adding an additional 441 μM of urea every 36 h. For comparison, the same supplementation protocols were tested in f/2 media, to help establish whether other (micro)nutrients beyond N were lacking in the NB. Two different sources of N was studied: 75 mg L^−1^ sodium nitrate (NaNO_3_) and 26 mg L^−1^ urea (CH_4_N_2_O), as shown in Table 2.

**Table 2:** *Halamphora* sp. cultivated in natural brine and f/2 minimal medium with urea supplementation

Sr no.	Conditions	Labels	Nitrogen source
1	f/2 medium with sodium nitrate	f/2 + NaNO3	sodium nitrate (NaNO3) (75 mg L−1)
2	natural brine without any additional supplementations	NB	–
3	f/2 medium with urea	f/2 + urea	urea (CH4N2O) (26 mg L−1)
4	natural brine with urea	NB + urea	urea (CH_4_N_2_O) (26 mg L^−1^)
5	f/2 medium with semi-continuous urea feed (SCUF)	f/2 + SCUF	urea (CH_4_N_2_O) (26 mg L^−1^)
6	natural brine with semi-continuous urea feed (SCUF)	NB + SCUF	urea (CH_4_N_2_O) (26 mg L^−1^)

The concentration of urea was selected based on previous studies by Rehmanji et al.^18^ and Acien et al.^20^

*Halamphora* sp. consumed approximately 26 mg L^−1^ of urea within 96 h (Figure 3A), with an average uptake rate of 0.18 mg L^−1^ h^−1^. Interestingly, this rate was comparable in both NB and f/2 medium, despite the absence of added macronutrients in the NB. This demonstrates that the NB likely contains sufficient trace elements to sustain basic cellular functions and support urea uptake in *Halamphora* sp. It is also possible the cells used internal nutrient reserves during the initial growth phase, allowing efficient urea assimilation.

**Figure 3:**
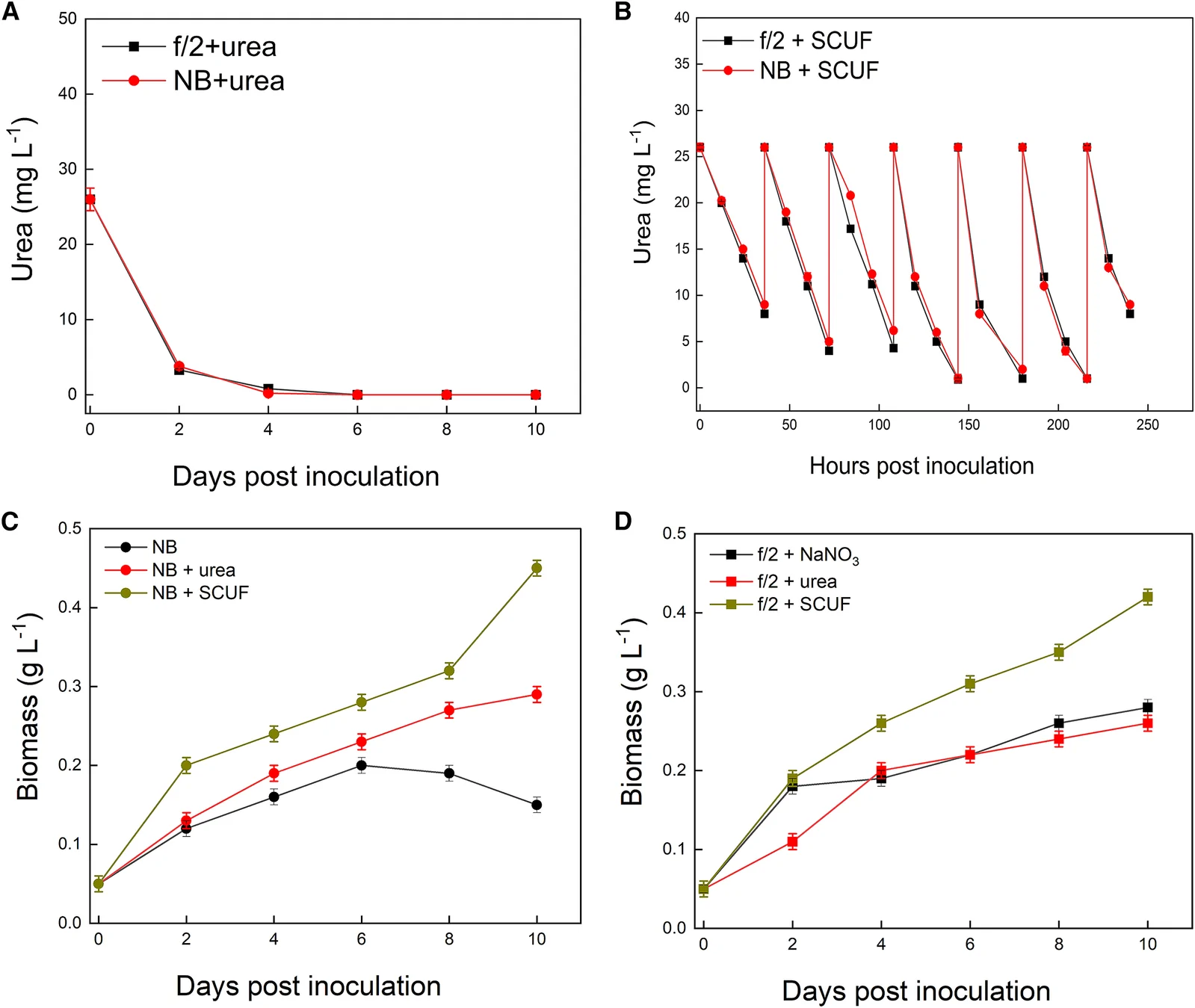
Growth responses and urea content of *Halamphora* sp. to supplementation with urea in natural brine and f/2 media over time (A) dissolved urea content in the f/2 medium (black square) and natural brine (NB) (red circle), where urea is added at the start with the inoculum with no further replenishment, (B) dissolved urea content in the f/2 medium (black square) and NB (red circle) where urea is added at equal intervals of 36 h making the process semi-continuous urea feed (SCUF), (C) biomass content of *Halamphora* sp. inoculated in NB (black circle), NB + urea at start (red circle) and NB + SCUF (green circle), (D) biomass content of *Halamphora* sp. inoculated in f/2 + NaNO_3_ (black square), f/2 + urea at start (red square) and f/2 + SCUF (green square). The starting inoculum density for this experiment was 0.05 g DCW L^−1^, concentration of NaNO_3_ was 75 mg L^−1^ and urea was 26 mg L^−1^. Data are represented as mean ± SD (standard deviation), *n* = 3 biological replicates. Student’s independent two-sample *t* tests (*df* = 4, two-tailed) were performed on the samples (*n* = 3 per condition) comparing treatment groups against the control. Significant values were determined on the basis of *p* < 0.0001.

Improved growth was observed in both NB (Figure 3C) and f/2 media (Figure 3D) with SCUF (Figure 3B), highlighting the effectiveness of urea as a nitrogen source for *Halamphora* sp. A single addition of urea to the NB also enhanced biomass production. By day 10, cultures with urea reached 0.28 g DCW L^−1^ (*p* < 0.01) biomass, whereas those without urea showed inhibited growth (Figure 3C). In SCUF, a maximum biomass of 0.45 g DCW L^−1^ (*p* < 0.01) was reached, nearly nine times greater than the starting inoculum, and 1.5 times higher than that achieved when urea was added once at the start of the experiment (0.29 g L^−1^). Additionally, SCUF regimen enhanced overall bioprocess selectivity, driving the (*SF*_Si/Li_) from 0 to >1,000 under fed-batch conditions (Table S5). Compared to a single batch urea addition (*SF*_Si/Li_ = 891). Overall, SCUF led to significantly enhanced growth and biomass production, corroborating earlier findings on marine diatoms such as *Phaeodactylum tricornutum* in SCUF condition.^18^

A critical consideration in diatom cultivation is nutrient cost, which contributes between 4% and 8% of the total biomass production expenses.^20^ A previous comparison of urea and sodium nitrate (NaNO3) as nitrogen sources confirmed that urea was more effective, producing the highest biomass yield at a lower substrate cost.^24^ Therefore, it may be possible to integrate nutrient recovery from secondary streams, such as urea from cow urine,^25^ which along with a semi-continuous feeding strategy could offer an economically viable and sustainable framework for diatom biomass cultivation with multiple applications.

### Effect of urea supplementation on Si removal and Li retention

The impact of SCUF on the Si removal efficiency was also evident. At an initial inoculum density of 0.05 g DCW L^−1^, *Halamphora* sp. in NB with after SCUF for 48 h (9 mg L^−1^), up to 97% of dissolved Si was removed (33.95 mg L^−1^) (*p* < 0.01). In contrast, in NB without any added urea, only 8% was removed (2.7 mg L^−1^) within 48 h, and in NB with urea added just at the start of the experiment, 54% of Si was removed (19 mg L^−1^) within 48 h (Figure 4A) (*p* < 0.01). The impact of SCUF on Si removal efficiency was profound. While the experiment was initiated with the same low inoculum density (0.05 g DCW L^−1^), the SCUF strategy sustained nutrient availability, preventing nitrogen depletion. This supported a rapid ∼4 times increase in biomass within 48 h in SCUF condition (Figure 3C NB + SCUF). Importantly, Li concentrations remained unaffected by urea supplementation (Figure 4B), indicating that the presence of urea or enhanced biomass had no adverse impact on Li retention in the brine. Intracellular hydrolysis of urea by urease generates ammonium, which is fixed via the glutamine synthetase-glutamate synthase (GS-GOGAT) pathway. This sustained nitrogen flux possibly maintains the continuous biosynthesis of frustule organic matrix components i.e., LCPAs and silaffins within SDVs. These biomolecules possibly catalyze polycondensation of monomeric silicic acid into amorphous silica frustules.

**Figure 4:**
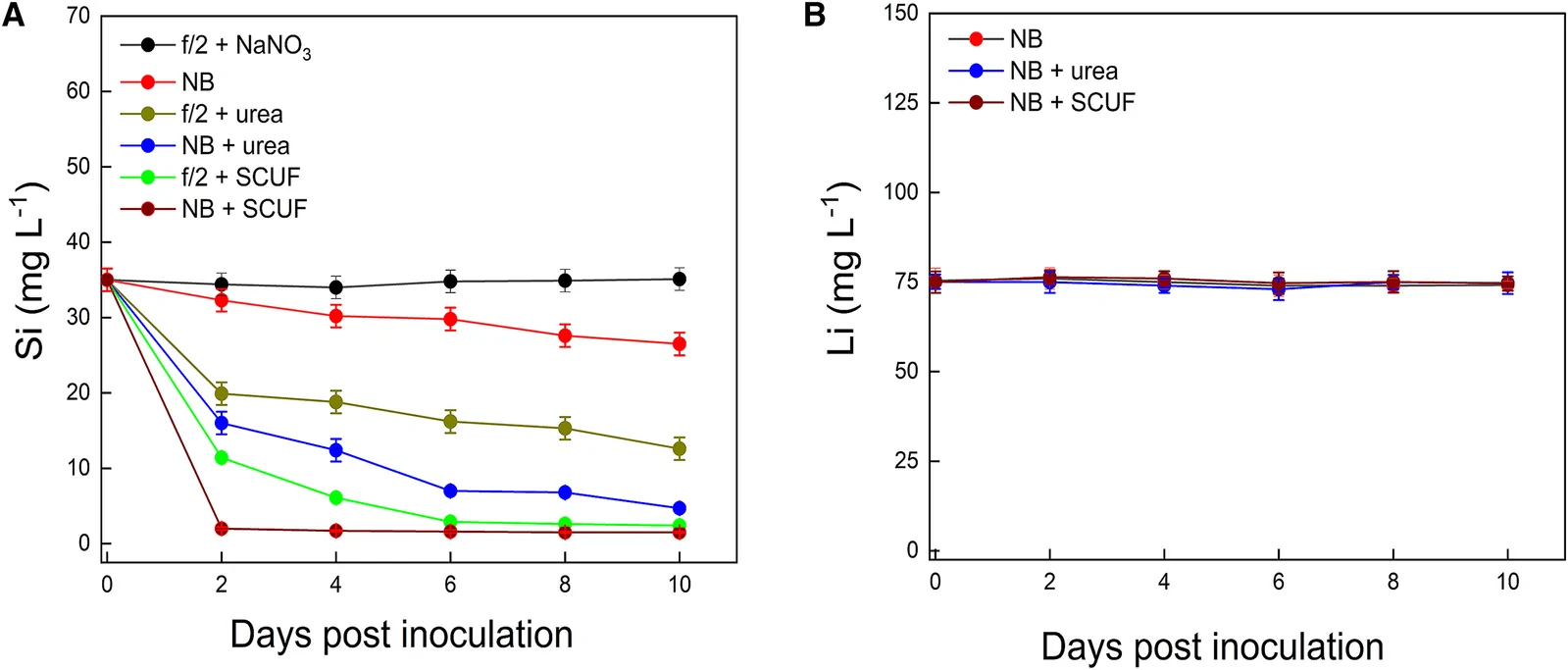
Si and Li concentrations in natural brine subjected to different conditions inoculated with *Halamphora* sp. The starting inoculum density for this experiment was 0.05 g DCW L^−1^. Conditions are f/2+ NaNO_3_ (black circles), NB (red circles), f/2+ urea (olive green circles), NB + urea (blue circles), f/2+ SCUF (green circles), and NB + SCUF (maroon circles). Plots show concentration of Si (A) and Li (B) in the solution over time. Data are represented as mean ± SD (standard deviation), *n* = 3 biological replicates. Student’s independent two-sample *t* tests (*df* = 4, two-tailed) were performed on the samples (*n* = 3 per condition) comparing treatment groups against the control. Significant values were determined on the basis of *p* < 0.0001.

These results open the possibility of using *Halamphora* sp. to remove Si from natural brines, which would reduce mineral scaling but also produce beneficial biomass that can be valorized further for high value biorenewables (see below). For example, a biotreatment system could involve feeding urea into natural brines that had been allowed to cool to temperatures suitable for growth and supplemented with *Halamphora* sp. and then stored in natural light for 48 h to allow Si removal. The brine could then go for downstream treatment for Li removal, and the biomass harvested for further applications. Further work would be required to realize this potential, including scale-up experiments and plant design.

### Effect of urea supplementation on FAME content of *Halamphora* sp.

Fatty acid methyl ester (FAME) analysis was undertaken to investigate whether diatom biomass recovered from NB processing could have commercial value as a source of high-value compounds such as omega-3 fatty acids. Results showed that the highest lipids yield

per liter were achieved in NB with SCUF, reaching 71 mg L^−1^ in 10 days (Table 3). This was largely due to the higher biomass achieved in these conditions, since the lipid content on a DCW basis was 16% DCW. In NB with only single urea feed much higher concentrations were achieved (21% DCW) and higher still in NB without urea supplementation (41 %DCW).

**Table 3:** Profiling of total fatty acid methyl esters (in %FAME %DCW, and mg L^−1^) using GC-MS in *Halamphora* sp. subjected to different growth conditions

Fatty acids	Conditions					
	f/2 + NaNO_3_		NB		f/2 + urea	
	% FAME	% DCW	% FAME	% DCW	% FAME	% DCW
C14	3.2 ± 0.05	0.7 ± 0.02	3.6 ± 0.13	1.5 ± 0.05	3.3 ± 0.12	0.7 ± 0.03
C14:1	0.9 ± 0.01	0.2 ± 0.01	0.8 ± 0.03	0.3 ± 0.01	0.8 ± 0.03	0.2 ± 0.01
C16	17.0 ± 0.40	3.6 ± 0.13	24.9 ± 0.87	10.2 ± 0.36	17.2 ± 0.60	3.8 ± 0.13
C16:1	27.9 ± 0.30	6.0 ± 0.21	32.1 ± 1.12	13.2 ± 0.46	25.1 ± 0.88	5.5 ± 0.19
C16:2	4.3 ± 0.20	0.9 ± 0.03	1.7 ± 0.06	0.7 ± 0.02	3.9 ± 0.14	0.8 ± 0.03
C16:3	8.4 ± 0.30	1.8 ± 0.06	4.0 ± 0.14	1.6 ± 0.06	7.2 ± 0.25	1.6 ± 0.06
C18	1.3 ± 0.05	0.3 ± 0.01	1.6 ± 0.06	0.6 ± 0.02	1.4 ± 0.05	0.3 ± 0.01
C18:1	4.5 ± 0.20	1.0 ± 0.03	8.7 ± 0.30	3.6 ± 0.12	5.4 ± 0.19	1.2 ± 0.04
C18:2	3.0 ± 0.10	0.6 ± 0.02	3.1 ± 0.11	1.3 ± 0.04	3.0 ± 0.10	0.7 ± 0.02
C18:3	3.1 ± 0.11	0.7 ± 0.02	2.6 ± 0.09	1.1 ± 0.04	2.9 ± 0.10	0.6 ± 0.02
C18:3	3.9 ± 0.14	0.8 ± 0.03	3.1 ± 0.11	1.3 ± 0.04	3.8 ± 0.13	0.8 ± 0.03
C18:4	3.8 ± 0.13	0.8 ± 0.03	2.4 ± 0.08	1.0 ± 0.03	3.9 ± 0.14	0.9 ± 0.03
C20:5	11.5 ± 0.40	2.5 ± 0.09	5.7 ± 0.20	2.3 ± 0.08	14.4 ± 0.50	3.2 ± 0.11
C22:6	7.4 ± 0.26	1.6 ± 0.06	5.7 ± 0.20	2.3 ± 0.08	7.7 ± 0.27	1.7 ± 0.06
SFA	21.4 ± 0.75	4.6 ± 0.16	30.1 ± 1.06	12.4 ± 0.43	21.9 ± 0.76	4.8 ± 0.17
MUFA	33.2 ± 1.16	7.1 ± 0.25	41.6 ± 1.46	17.1 ± 0.60	31.3 ± 1.10	6.9 ± 0.24
PUFA	45.4 ± 1.59	9.7 ± 0.34	28.2 ± 0.99	11.6 ± 0.40	46.8 ± 1.64	10.2 ± 0.36
Total FAMEs	**titer (mg L**^**−**^**^1^)**	**content (% DCW)**	**titer (mg L**^**−**^**^1^)**	**content (% DCW)**	**titer (mg L**^**−**^**^1^)**	**content (% DCW)**
	60.0 ± 0.67	21.0 ± 0.43	61.0 ± 0.33	41.0 ± 0.66	57.0 ± 0.21	30.0 ± 0.12

Fatty acids	Conditions					
	NB + urea		f/2 + SCUF		NB + SCUF	
	% FAME	% DCW	% FAME	% DCW	% FAME	% DCW
C14	2.4 ± 0.08	0.5 ± 0.02	2.5 ± 0.09	0.4 ± 0.01	3.4 ± 0.12	0.5 ± 0.02
C14:1	0.4 ± 0.01	0.1 ± 0.00	0.4 ± 0.02	0.1 ± 0.00	0.7 ± 0.02	0.1 ± 0.00
C16	18.6 ± 0.65	4.0 ± 0.14	12.4 ± 0.43	1.9 ± 0.07	9.4 ± 0.33	1.5 ± 0.05
C16:1	20.8 ± 0.73	4.4 ± 0.16	14.1 ± 0.49	2.2 ± 0.08	12.7 ± 0.44	2.0 ± 0.07
C16:2	2.2 ± 0.08	0.5 ± 0.02	3.6 ± 0.13	0.5 ± 0.02	1.9 ± 0.07	0.3 ± 0.01
C16:3	5.0 ± 0.18	1.1 ± 0.04	7.4 ± 0.26	1.1 ± 0.04	3.4 ± 0.12	0.5 ± 0.02
C18	2.2 ± 0.08	0.5 ± 0.02	2.0 ± 0.07	0.3 ± 0.01	2.6 ± 0.09	0.4 ± 0.01
C18:1	7.6 ± 0.27	1.6 ± 0.06	5.8 ± 0.20	0.9 ± 0.03	10.5 ± 0.37	1.7 ± 0.06
C18:2	5.7 ± 0.20	1.2 ± 0.04	2.7 ± 0.09	0.4 ± 0.01	1.7 ± 0.06	0.3 ± 0.01
C18:3	6.1 ± 0.21	1.3 ± 0.05	3.5 ± 0.12	0.5 ± 0.02	2.2 ± 0.08	0.3 ± 0.01
C18:3	4.5 ± 0.16	1.0 ± 0.03	4.7 ± 0.17	0.7 ± 0.03	4.8 ± 0.17	0.8 ± 0.03
C18:4	3.1 ± 0.11	0.7 ± 0.02	4.1 ± 0.14	0.6 ± 0.02	1.9 ± 0.07	0.3 ± 0.01
C20:5	11.3 ± 0.39	2.4 ± 0.08	26.2 ± 0.92	4.0 ± 0.14	30.1 ± 1.05	4.7 ± 0.17
C22:6	10.1 ± 0.35	2.2 ± 0.08	10.5 ± 0.37	1.6 ± 0.06	14.8 ± 0.52	2.4 ± 0.08
SFA	23.3 ± 0.82	5.0 ± 0.17	16.8 ± 0.59	2.6 ± 0.09	15.4 ± 0.54	2.4 ± 0.08
MUFA	28.8 ± 1.01	6.2 ± 0.22	20.4 ± 0.71	3.1 ± 0.11	23.8 ± 0.83	3.8 ± 0.13
PUFA	47.9 ± 1.68	10.2 ± 0.36	62.8 ± 2.20	9.6 ± 0.34	60.8 ± 2.13	9.6 ± 0.34
Total FAMEs	**titer (mg L**^**−**^**^1^)**	**content (% DCW)**	**titer (mg L**^**−**^**^1^)**	**content (% DCW)**	**titer (mg L**^**−**^**^1^)**	**content (% DCW)**
	62.0 ± 0.69	21.0 ± 0.16	64.0 ± 0.23	15.0 ± 0.11	71.0 ± 0.13	16.0 ± 0.21

Data are represented as mean ± SD (standard deviation), *n* = 3 biological replicates. Student’s independent two-sample *t* tests (*df* = 4, two-tailed) were performed on the samples (*n* = 3 per condition) comparing treatment groups against the control. Significant values were determined on the basis of *p* < 0.0001. The bold value are headings for total FAME content. FAME content is represented in % FAME, %DCW, Titre (mg/L) and content (DCW%) as recommended by reviewers.

This high lipid accumulation under nutrient-limited conditions (specifically, the absence of added nitrogen) is a well-documented stress response in diatoms and suggests that biomass from the brine remediation process itself, even before optimization with urea, is lipid-rich and could be further investigated for biofuel application.

*Halamphora* sp. cultivated in NB without urea exhibited markedly higher proportions of C16 (10% DCW), C16:1 (13% DCW), and C18:1 (3.6% DCW) (Table 3). In contrast, cultures supplemented with urea at the start showed reduced levels of these components, with C16, C16:1, and C18:1 accounting for 4%, 4.4%, and 1.6% DCW, respectively. A further decline was observed under SCUF, where the respective contributions decreased to 1.5%, 2.0%, and 1.7% DCW. These fatty acids are considered favorable for biodiesel applications due to their oxidative stability and desirable cold-flow properties.^26^ This enhanced lipid accumulation aligns with previous findings that showed that nitrogen deficiency can trigger the production of storage lipids such as triacylglycerols (TAGs) in diatoms.^27^^,^^28^^,^^29^ Collectively, the FAME analysis reveals that nitrogen deprivation (i.e., cultivation without urea) steers the lipid profile of *Halamphora* sp. toward compositions suitable for biofuel applications. This underscores the significant commercial potential of the biomass co-produced during Si removal from brine, allowing the process to be optimized for biofuel feedstocks production, thereby enhancing the overall economic viability of the bioremediation workflow.

### Effect of urea supplementation on EPA and DHA production in natural brine via *Halamphora* sp.

The production of EPA and DHA by *Halamphora* sp. was quantified under different culture conditions (Figure 5). Across all tested conditions, both volumetric yields (mg L^−1^) and the absolute cellular content (% DCW) of EPA exhibited a progressive increase over time, indicating sustained fatty acid biosynthesis during growth (Figures 5A and 5B). By contrast similar trend was observed for volumetric yields (mg L^−1^) of DHA.

**Figure 5:**
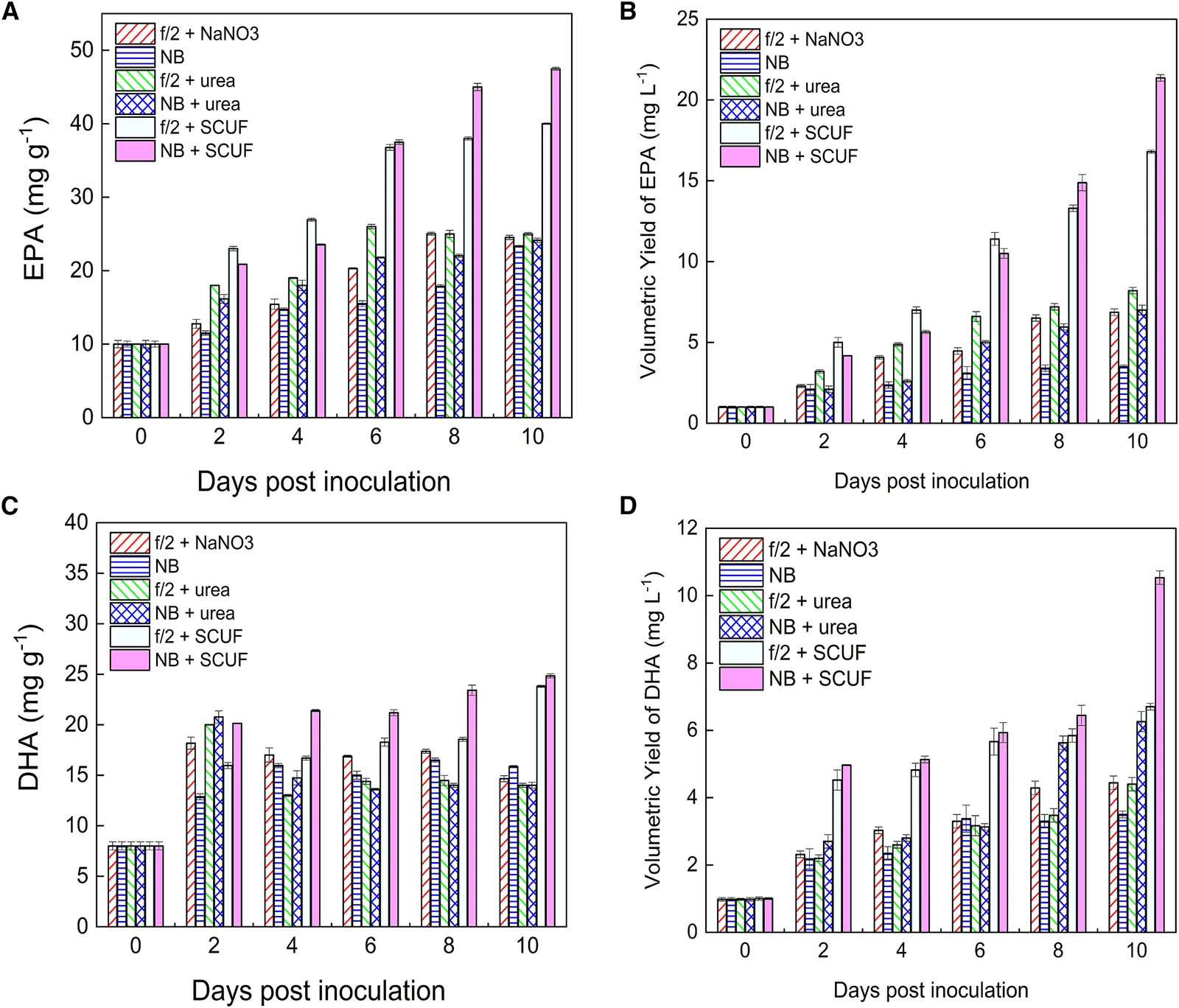
Eicosapentaenoic acid and Docosahexaenoic acid quantification in different conditions inoculated with *Halamphora* sp. conditions are f/2+ NaNO_3_ (red), NB (blue), f/2+ urea (green), NB + urea (blue cross), f/2+ SCUF (white), and NB + SCUF (pink) Plots show EPA content (A), and EPA yield (B), DHA content (C), and DHA yield (D) in the solution over time. Data are represented as mean ± SD (standard deviation), *n* = 3 biological replicates. Student’s independent two-sample *t* tests (*df* = 4, two-tailed) were performed on the samples (*n* = 3 per condition) comparing treatment groups against the control. Significant values were determined on the basis of *p* < 0.0001.

The rate of this increase was strongly influenced by the cultivation strategy. The highest EPA accumulation was consistently observed under NB + SCUF and f/2 + SCUF conditions, which outperformed all other treatments at each sampling point (Figures 5A and 5B). In particular, under NB + SCUF, EPA content increased from 10 to 47.5 mg g^−1^ over 10 days, representing the highest final concentration among all conditions. This enhanced accumulation under SCUF is consistent with previous findings demonstrating that continuous nitrogen supplementation promotes EPA biosynthesis in diatom.^24^ A similar pattern was observed for DHA production. The NB + SCUF treatment resulted in the highest accumulation, reaching a maximum of 23.5 mg g^−1^ by day 10 (Figures 5C and 5D), confirming the positive effect of sustained urea feeding on DHA production. The rapid Si removal observed after 48 h and the later peak in EPA and DHA accumulation after 10 days reflect two distinct phases of the bioprocess. The early phase is driven by active Si uptake and frustule formation during rapid growth, whereas omega-3 biosynthesis continues over the longer cultivation period as biomass accumulates. Therefore, the system is best considered as a two-stage process, with an initial short brine pretreatment stage for Si removal and Li retention followed by a biomass valorization stage for EPA and DHA production.

EPA and DHA are key membrane lipids typically associated with growth-supporting conditions and are considered high-value compounds due to their essential roles in human health and strong market demand.^30^ The global omega-3 ingredients market exceeded USD 1 billion in 2025 and is projected to grow at a compound annual growth rate (CAGR) of over 13.8% between 2025 and 2030.^31^ These long-chain omega-3 PUFAs play critical roles in cardiovascular, neurological, and anti-inflammatory functions, yet cannot be synthesized efficiently by humans.^32^ As traditional fish-derived sources face increasing pressure from declining stocks, microalgae particularly diatoms have emerged as a sustainable and vegetarian alternative for the production of these nutraceuticals.^33^

Maintaining elevated EPA and DHA levels requires adequate nitrogen availability to balance cellular growth and metabolite production. The enhanced EPA and DHA accumulation observed under SCUF supplementation can therefore be attributed to increased nitrogen availability, which supports membrane lipid biosynthesis in diatoms. This interpretation is consistent with previous reports demonstrating elevated EPA accumulation under nitrogen-enhanced conditions.^24^ Importantly, the ability to generate high-value omega-3 fatty acids while simultaneously bioprocessing silica-rich geothermal brines presents a compelling opportunity to improve overall resource efficiency. Repurposing the biomass generated during Si removal as a valuable feedstock reduces waste generation and enhances the environmental footprint of the process. Overall, these findings demonstrate that *Halamphora* sp. is well suited for integration into multiproduct biorefinery systems, where Si mitigation from geothermal brines can be coupled with the sustainable production of high-value omega-3 PUFAs for use in aquaculture, pharmaceutical, or nutraceutical applications, assuming the necessary regulatory and safety standards can be met.

### Preliminary mass balance, theoretical co-product valuation, and limitations of the bioprocess

To evaluate the material requirements and potential co-product yield of the proposed biological pre-treatment, a preliminary mass-balance estimate was conducted based on bench-scale performance. Preprocessing of 1,000 L of natural geothermal brine via SCUF diatom-based approach requires 50 g DCW biomass of *Halamphora* sp. (initial inoculum density: 0.05 g L^−1^). The total urea required across six semi-continuous feedings (SCUF) amounts to approximately 200 g (∼£3 at commercial agricultural grades). Post treatment 0.5 kg dry biomass is produced which contains approximately 25 g of EPA and 12 g of DHA (37 g total LC-PUFA), the values obtained are comparable to those reported in previous studies. Additionally, the process retains >99% of dissolved Li in the liquid phase for downstream extraction. Applying bulk, unrefined lipid market estimates a theoretical raw co-product credit of ∼£1.10 per 1,000 L of processed brine, prior to downstream processing is obtained. This creates an opportunity to serve as a secondary revenue generated.

However, this preliminary valuation serves only as a baseline mass-balance estimate and excludes significant operational and capital expenditures (OPEX and CAPEX). A comprehensive economic evaluation must incorporate downstream processing costs, including brine cooling, culture mixing and illumination, cell harvesting, lipid extraction, and purification. Furthermore, geo-chemical analysis indicated that trace metals largely remained in solution (Figure S1); cultivating biomass in mineral-rich geothermal fluids introduces regulatory and purity constraints that require further validation. Therefore, while these findings establish the technical feasibility of dual Si removal and Li preservation, further analysis is necessary to determine industrial scalability and net economic viability.

This study establishes a bioprocess using the marine diatom *Halamphora* sp. for the simultaneous treatment of geothermal brine and the production of high-value bioproducts. Compared to bacterial MICP approaches for pre-treatment of identical Cornish geothermal brines^25^ this diatom-based system offers 2 key advancements: First, it targets Si removal (97% Si reduction within 48 h), which remains unaddressed by MICP. Second, while post MICP treatment it yields mineral slurries suitable as agricultural soil amendments, diatom biomass yields high-value PUFAs (47.5 mg g^−1^ EPA and 23.5 mg g^−1^ DHA).

Additionally compared to conventional industrial silica-mitigation methods for pre-processing of geothermal brines, the diatom-based approach offers several distinct operational, chemical, and economic advantages (Table S6). Together, these distinct biological systems could form a sequential, multi-barrier pre-treatment pipeline where stage 1 is diatom-based Si removal and stage 2 is bacterial based MICP treatment for Ca-Mg removal, prior to DLE. Further the implementation of a (SCUF) strategy was pivotal, enhancing biomass production by 9-fold and dramatically accelerating Si uptake by sustaining active growth. This cultivation approach strategically steers the lipid metabolism of *Halamphora* sp., enabling the co-production of nutraceuticals. By effectively coupling contaminant removal (Si) with resource retention (Li) and the synthesis of valuable omega-3 fatty acids, this work presents a circular bioeconomy model. It moves beyond single-output bioremediation, offering a scalable and economically attractive strategy for the sustainable processing of geothermal resources.

### Limitations of the study

The limitations of our study are that the proposed *Halamphora* sp.-assisted bioprocess was evaluated using a single natural geothermal brine under laboratory-scale conditions. Therefore, its robustness should be tested across a wider range of geothermal brines with varying chemical compositions, including differences in salinity, pH, Si concentration, trace metals, and competing ions. Further Li recovery was not directly evaluated using a DLE process after biological pretreatment. These limitations can be addressed by screening brine-diversity, direct coupling with Li extraction systems, and techno-economic and life cycle assessments to evaluate the scalability, product quality, and overall feasibility of the proposed bioprocess.

## Resource availability

### Lead contact

Requests for further information and resources should be directed to and will be fulfilled by the lead contact, Mohammed Rehmanji (m.rehmanji@exeter.ac.uk).

### Materials availability

This study did not generate new unique reagents.

### Data and code availability

• Raw data from Figures 1, 2, 3, and 4 have been deposited at Mendeley Data and are publicly available at https://doi.org/10.17632/z97vm778wy.1.
• This paper does not report original code.
• Any additional information required to reanalyze the data reported in this paper is available from the lead contact upon request.

## Acknowledgments

This work was supported by 10.13039/501100000268BBSRC through the grant Bioprocessing of Lithium Brines (BB/X011658/1) and by NERC through the grant Lithium for Future Technology: LiFT (NE/V007009/1). The research data supporting this publication are provided within this paper and in the supplementary information accompanying this paper. We thank members of the Cornish Geothermal Project for providing natural brine solution and at the University of Exeter, Gavyn Rollinson for assistance with SEM analysis and Cristopher Mitchell for guidance with GC-MS analysis. For the purpose of open access, the author has applied a “Creative Commons Attribution (CC BY)” license to any author accepted manuscript version arising from this submission.

## Author contributions

Conceptualization, M.R., K.A.H.-E., L.N., and A.S.; methodology, M.R., K.A.H.-E., and L.N.; investigation, M.R., A.S., and L.N.; writing – original draft, M.R.; writing – review and editing, M.R., K.A.H.-E., L.N., and A.S.; funding acquisition, L.N., A.S., and K.A.H.-E.; resources, L.N., A.S., and K.A.H.-E.; supervision, L.N., A.S., and K.A.H.-E.

## Declaration of interests

The authors declare no competing interests.

## STAR★Methods

### Key resources table

**Table:** 

REAGENT or RESOURCE	SOURCE	IDENTIFIER
**Chemicals**		

Nutrients added to artificial sea water (ASW)		
Sodium nitrate	Fisher Scientific, UK	7631-99-4
Sodium dihydrogen phosphate dihydrate	Fisher Scientific, UK	13472-35-0
Sodium metasilicate nonahydrate	Fisher Scientific, UK	13517-24-3

**Artificial sea water (ASW)**		

Sodium chloride	Fisher Scientific, UK	7647-14-5
Magnesium chloride hexahydrate	Fisher Scientific, UK	7791-18-6
Sodium sulfate	Fisher Scientific, UK	7757-82-6
Calcium chloride hexahydrate	Fisher Scientific, UK	7774-34-7
Potassium bromide	Fisher Scientific, UK	7758-02-3
Boric acid	Fisher Scientific, UK	10043-35-3
Sodium metasilicate nonahydrate	Fisher Scientific, UK	13517-24-3
Strontium chloride hexahydrate	Fisher Scientific, UK	10025-70-4
Sodium fluoride	Fisher Scientific, UK	7681-49-4
Ammonium nitrate	Fisher Scientific, UK	6484-52-2
Iron (III) phosphate tetrahydrate	Fisher Scientific, UK	4684-52-2

**Trace-metal solution**		

Zinc sulfate heptahydrate	Fisher Scientific, UK	7446-20-0
Manganese (II) sulfate monohydrate	Fisher Scientific, UK	10034-96-5
Sodium molybdate dihydrate	Fisher Scientific, UK	10102-40-6
Cobalt (II) sulfate heptahydrate	Fisher Scientific, UK	10026-24-1
Copper (II) chloride dihydrate	Fisher Scientific, UK	10125-13-0
Ammonium iron (II) sulfate hexahydrate	Fisher Scientific, UK	7783-85-9
Disodium ethylenediaminetetraacetate dihydrate	Fisher Scientific, UK	6381-92-6

**Vitamins**		

Cyanocobalamin (vitamin B_12_)	Fisher Scientific, UK	68-19-9
Biotin (vitamin B_7_)	Fisher Scientific, UK	58-85-5
Thiamine (vitamin B_1_)	Fisher Scientific, UK	67-03-8

**Deposited data**		

Raw data files	This paper	https://doi.org/10.17632/z97vm778wy.1

**Experimental models: Organisms/strains**		

*Conticribra weissflogii*	CCAP	1085/18
*Fistulifera pelliculosa*	CCAP	1050/9
*Halamphora* sp.	CCAP	1031/1
*Thalassiosira pseudonana*	CCAP	1085/12P
Swan Pool Diatom Isolate	Natural site Cornwall, Swan pool lake.	NA
Kennall Vale Diatom Isolate	Natural site Cornwall, Kennall Vale Nature Reserve	50°20′ N, 5°.2′ W

**Software**		

SEM analysis: xT microscope control 6.2.7	Thermo Fisher Scientific	v6.2.7
GC-MS analysis: MassHunter software	Agilent	vB.12.00
IC analysis: Chromeleon 7.0 software	Thermo Fisher Scientific	v7.0

**Other**		

Scanning Electron Microscope	Thermo Scientific	FEI Quanta FEG 650 SEM-EDS
Inductively Coupled Plasma Optical Emission Spectroscopy	Agilent	Agilent 5110 VDV
Ion Chromatography	Thermo Fischer Scientific	Dionex IonPac AS11-HC
Gas Chromatography	Agilent	Agilent 7250

### Experimental model and study participant details

Four marine diatom strains were selected to investigate their potential for Si removal based on their distinct physiological traits and ecological relevance. *Conticribra weissflogii* CCAP 1085/18, a large centric diatom known for its high Si assimilation capacity under variable nutrient conditions.^34^ *Fistulifera pelliculosa* CCAP 1050/9, a small pennate diatom known for its rapid growth, was included due to its robust performance in saline environments and ability to accumulate significant silica within its frustules.^35^ *Halamphora* sp. CCAP 1031/1, a benthic diatom capable of tolerating fluctuating salinity and nutrient levels was selected to assess Si uptake under stress conditions.^22^ *Thalassiosira pseudonana* CCAP 1085/12P, a well-established model species with a fully sequenced genome and well-characterized Si metabolism, served as a genetic and physiological benchmark.^36^ All strains were obtained from the Culture Collection of Algae and Protozoa (CCAP, https://www.ccap.ac.uk). The diatoms were maintained with f/2 nutrients in artificial sea water, pH ∼7.2, prepared as by Shaikh et al., 2019 and autoclaved at 121 °C for 20 min before use. Cultures were maintained at 24 °C, 110 rpm, 35 μmol m^−2^ s^−1^ photosynthetically active radiation (PAR) illumination under a light regime of 16:8 in orbital type Infors HT Multitron PRO Triple Stack Incubator Shaker (UK).

Along with the four model diatoms, two local strains were isolated from samples collected at the freshwater river at Kennall Vale Nature Reserve (50°20′ N, 5°.2′ W) and the tidal Swanpool Lake, both located in Cornwall, UK. Diatoms were isolated by spreading samples on 1.5% agar plate (f/2 made in 50% artificial sea water). Plates were exposed to 60 μmol m^−2^ s^−1^ illumination under a light regime of 16:8, at 24 °C in Infors HT Multitron PRO Triple Stack Incubator (UK). After 20 days a single brown colony was picked from individual plates and inoculated in 10 mL of autoclaved water collected from the natural sites amended with f/2 nutrients. In subsequent passages this autoclaved water was replaced with artificial sea water. Growth was measured as change in dry weight.^37^ For each measurement, 15 mL of cell culture was centrifuged at 3200 g in pre-weighed tubes, the pellet was dried at 60 °C for 48 h before re-weighing the tube.

### Method details

#### Natural brine (NB) and Si removal experiments

To test the hypothesis that diatoms can be used to remove Si from Li-bearing geothermal brine and to screen the diatom species required for performing Si removal, a natural brine was obtained from a Cornish geothermal project (Cornwall, south-west England, UK). The composition of this natural brine was determined using an Agilent 5110 VDV Inductive Coupled Plasma – Optical Emission Spectrometer (ICP-OES), using the method and parameters as described by Rehmanji et al.^25^ The brine was sterilized by autoclaving at 121 °C for 20 min before the experiments and no nutrients were added.

To evaluate the biological selectivity of biosilicification for silicon removal relative to lithium, the Silicon-Lithium Separation Factor (SF_Si/Li_) was calculated according to Equation 1:^38^

(Equation 1) $$SF_{\text{Si}/\text{Li}}=\frac{\left({\left[\text{Si}\right]}_{i}-{\left[\text{Si}\right]}_{f}\right)/{\left[\text{Si}\right]}_{f}}{\left({\left[\text{Li}\right]}_{i}-{\left[\text{Li}\right]}_{f}\right)/{\left[\text{Li}\right]}_{f}}$$

where [Si]_i_ and [Si]_f_ represent the initial and final Si concentrations (mg L^−1^), and [Li]_i_ and [Li]_f_ represent the initial and final Li concentrations (mg L^−1^) in the natural brine, respectively. Separation factor above unity is in favor of Si uptake over Li.

The six diatom species (*C. weissflogii*, *F. pelliculosa*, *Halamphora* sp., *T. pseudonana*, Kennall Vale isolate and Swanpool isolate) were grown to the mid-logarithmic phase (day 5), harvested by centrifugation (3200 *g*, 15 min), washed using a 1X phosphate buffered saline solution, centrifuged again, then inoculated into 100 mL NB solution at a starting dry biomass concentration of 0.5 g DCW L^−1^. A flask without any diatom biomass was used as a control. To evaluate the optimal cell concentration required for performing Si removal, mid-logarithmic phase cells from the primary culture of *Halamphora* sp. were harvested by centrifugation (3200 *g*, 15 min), washed using phosphate buffered saline, centrifuged again, and inoculated into the natural brine solution in standard 250 mL conical flasks containing 100 mL of culture volume. The initial biomass concentration was varied from 0.05, 0.1, 0.25, 0.5, 1.0 g DCW L^−1^, with controls including f/2 with and without added biomass. The initial dissolved Si concentration was standardized across all experimental treatments. While the natural brine contained an inherent Si concentration of 35 mg L^−1^, the f/2 medium was modified by adding 30 mg L^−1^ of Si this enable a direct comparison across the experimental condition. A full silicon mass balance, biomass digestion for biomass associated Si quantification for all the conditions, and killed-cell/dark controls were not performed in this study but could be addressed in future work.

#### Urea supplementation experiments

To investigate the effect of urea supplementation, *Halamphora* sp. was cultured in f/2 and natural brine with two different sources of N added: 75 mg L^−1^ sodium nitrate (NaNO_3_) or 26 mg L^−1^ urea (CH_4_N_2_O) (Table 2). The mid-logarithmic phase culture (day 4, with initial density of 0.05 g DCW L^−1^ biomass) was inoculated into 150 mL medium in 500 mL Erlenmeyer flasks. To assess the effect of urea supplementation on Si uptake and biomass production, urea was added to the flasks containing natural brine at defined intervals. Urea supplementation under SCUF was applied at 36, 72, 108, 144, 180, and 216 h after inoculation (Table S1). At each feeding, urea was restored to 26 mg L^−1^ (441 μM). In a 150 mL working volume, this corresponds to a nominal cumulative addition of 182 mg L^−1^ including the initial inoculation dose. Consumption rates were determined by measuring residual urea concentrations at fixed time points during preliminary tests (Table S1). The starting inoculum density was 0.05 g DCW L^−1^; this was selected based on the results of screening of different biomass density for Si uptake. Starting with a lower density allows for a more robust demonstration of the culture’s growth potential and nutrient assimilation efficiency, as it avoids early-onset nutrient limitation and ensures that high biomass production and rapid Si uptake are achieved through active growth rather than being a function of a high starting biomass.

#### SEM analysis

To characterize the surface morphology of *Halamphora* sp., 2 mL of cells grown in natural brine without additional N for 10 days, was centrifuged (3200 *g*, 15 min) and the cell pellet was washed three times with phosphate buffer saline. After washing, the pellet was fixed in 1 mL of 2.5% glutaraldehyde solution overnight and then centrifuged (3200 *g*, 5 min) to remove the residual glutaraldehyde. The samples were further treated with 1 mL of 1.5% glutaraldehyde for 30 min, centrifuged (3200 *g*, 15 min) and finally treated with 1 mL of 0.75% glutaraldehyde as described in.^39^ Subsequently, cells were sequentially dehydrated with ethanol (30%, 40%, 50%, 60%, 70%, 80%, 90% and 100%) for 30 min at each concentration.^25^ The 100% ethanol samples were loaded on clean silicon wafers attached to aluminum stubs and left to dry in air at room temperature (23 °C ± 2 °C). Images of the samples were obtained using a FEI Quanta FEG 650 SEM-EDS using operating conditions as described in Table S2. We acknowledge that SEM-EDS mapping for all the conditions was not performed in this study but could be addressed in future work.

#### Quantification of brine components and urea

Concentrations of Na, K, Li, Ca, Mg, Sr, B, Cu, Fe, Mn, Si and Ni in the natural brine solution were determined using an Agilent 5110 VDV Inductive Coupled Plasma – Optical Emission Spectrometer (ICP-OES). The brine solution was filtered and diluted (1:1000) into 2% nitric acid (HNO_3_). All reagents used were analytical grade. Measurements were made in axial view mode using wavelengths and operating parameters are presented in Table S3.

For accurately measuring silicon (Si) by ICP-OES, a multi-point calibration curve was prepared using a certified Si standard solution [Thermo Fisher Scientific] diluted 100 times in 2% HNO_3_. The calibration curve exhibited linearity over the measured concentration range, with a correlation coefficient (R^2^) of 0.9998. Solutions containing 2% HNO_3_ were analyzed as blank with each batch of samples to account for any background contamination. For prevention of Si contamination from glassware, all sample preparation steps including dilution, storage, and standard preparation were conducted using clean plasticware (polypropylene tubes and bottles). The detection wavelength used for measuring Si content was 288.158 nm. For anion analysis the natural brine was passed through 0.22 μm filters, diluted using deionized water and analyzed using a high-pressure ion chromatography system (Dionex Integrion HPIC, Thermo Fischer Scientific) equipped with a Dionex IonPac AS11-HC column (2 mm × 250 mm, 4 μm particle size) and a 10 μL injection loop. Chromeleon 7.0 software was used for data processing and cation; anion concentrations were measured using standard calibrations. The instrumental parameters are listed in Table S4.

Urea concentrations were estimated using a spectrophotometric assay described by ref.^40^ Briefly, experimental samples were centrifuged (3200 g, 15 min) and 50 μL of supernatant was transferred into a clear flat-bottom 96-well plate. The working reagent consisted of *o*-phthalaldehyde (100 mg L^−1^), primaquine bisphosphate (513 mg L^−1^), sulfuric acid (2.5 M), boric acid (2.5 g L^−1^), and Brij-35 (0.03%). Two hundred μL of freshly prepared working reagent were added to the supernatant and mixed gently on a shaker for 1 h at 20 ± 2 °C. The optical density at 430 nm was measured and results were compared to a calibration curve made using dilutions of a 50 mg L^−1^ of commercial urea.

#### Quantification of total fatty acid methyl esters (FAMEs)

A modified Bligh-dyer technique was adapted from ref.^37^ for extracting total FAMEs. Briefly 25 mg of cells sample were collected on days 0, 2, 4, 6, 8, and 10 from individual flasks. The samples were centrifuged at 3200 g for 15 min and the pellet was subjected to acid-hydrolysation and methyl-esterification using 2% sulfuric acid in methanol (300 μL) for 2 h at 80 °C. Prior to the reaction, 50 μg of nonadecanoic acid (Alfa Aesar) was added as internal standard. FAME Mix (Supelco) was used as reference standard mixture for the fatty acid identification (Figure S3). The fatty acid methyl esters were extracted using 300 μL of 0.9% (w/v) NaCl solution and 300 μL of hexane. The mixture was vortexed briefly and centrifuged at 3000 × *g* for 3 min to separate the phases. Two microlitre of hexane layer was injected into an Agilent 7250 gas chromatography (GC) machine with a high-resolution quadrupole time-of-flight (Q-ToF) mass spectrometer, capable of using both electron Ionization (EI) and chemical Ionization (CI), it was connected with an OMEGAWAX 250 column (30 m × 0.25 m × 0.25 m). The GC-MS running conditions were as stated by Shaikh et al. (2019) The samples were run in MS in the split mode (1:5) for 25 min, with the beginning oven temperature set at 150 °C and a simultaneous escalation of 10 °C min^−1^ to 240 °C. For quenching, helium (He) and nitrogen (N_2_) gas were employed at flow rates of 2.25 and 1.5-mL min^−1^, respectively. The data were collected using the quadrupole selective mass detector, which has a scan range of 30–550 amu. Further MassHunter software,^41^ was used to search GC-MS spectra against the NIST library, applying an R value threshold of 600.

### Quantification and statistical analysis

All experimental treatments were performed in biological triplicates (*n* = 3), and dataset values are presented as mean ± SD. Differences were evaluated using independent two-tailed Student’s t-tests. Statistical significance was established at *α* = 0.05. All statistical evaluations and calculations were performed using Origin (version 10.0) and Python (SciPy library).
