# Supplementary material for: Diatom-based treatment of geothermal brines to selectively remove silicon, retain lithium for downstream recovery, and produce omega-3 fatty acids
Source: iScience. 2026 Oct 26;29(11):117965. doi: 10.1016/j.isci.2026.117965 (PMC13735000; doi:10.1016/j.isci.2026.117965)
Supplement: Supplementary file 1 [file mmc1.pdf]

iScience, Volume *29*

Supplemental information

Diatom-based treatment of geothermal brines to selectively remove silicon, retain lithium for downstream recovery, and produce omega-3 fatty acids

Mohammed Rehmanji, Alastair Skeffington, Karen A. Hudson-Edwards, and Laura Newsome

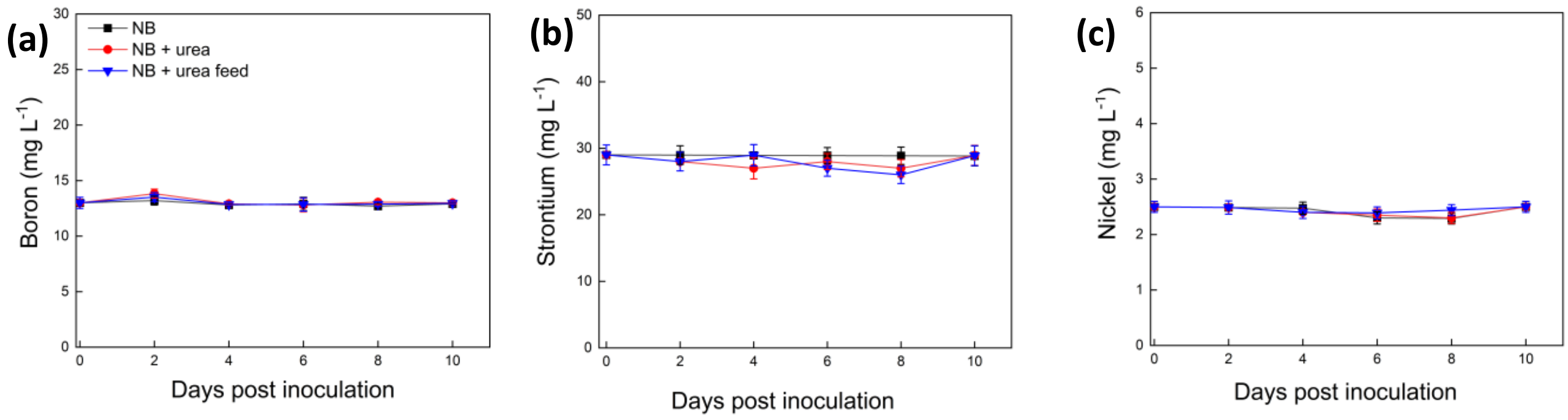

Fig S1. Metal concentrations in natural brine inoculated with *Halamphora* sp. natural brine without cells (black squares), Natural brine with urea (red circles) and Natural brine + SCUF (Blue inverted triangle). Plots show concentration of B (a), Sr (b) and Ni (c) measured in the solution over time. Data are represented as mean ± SD (standard deviation), n = 3 biological replicates.

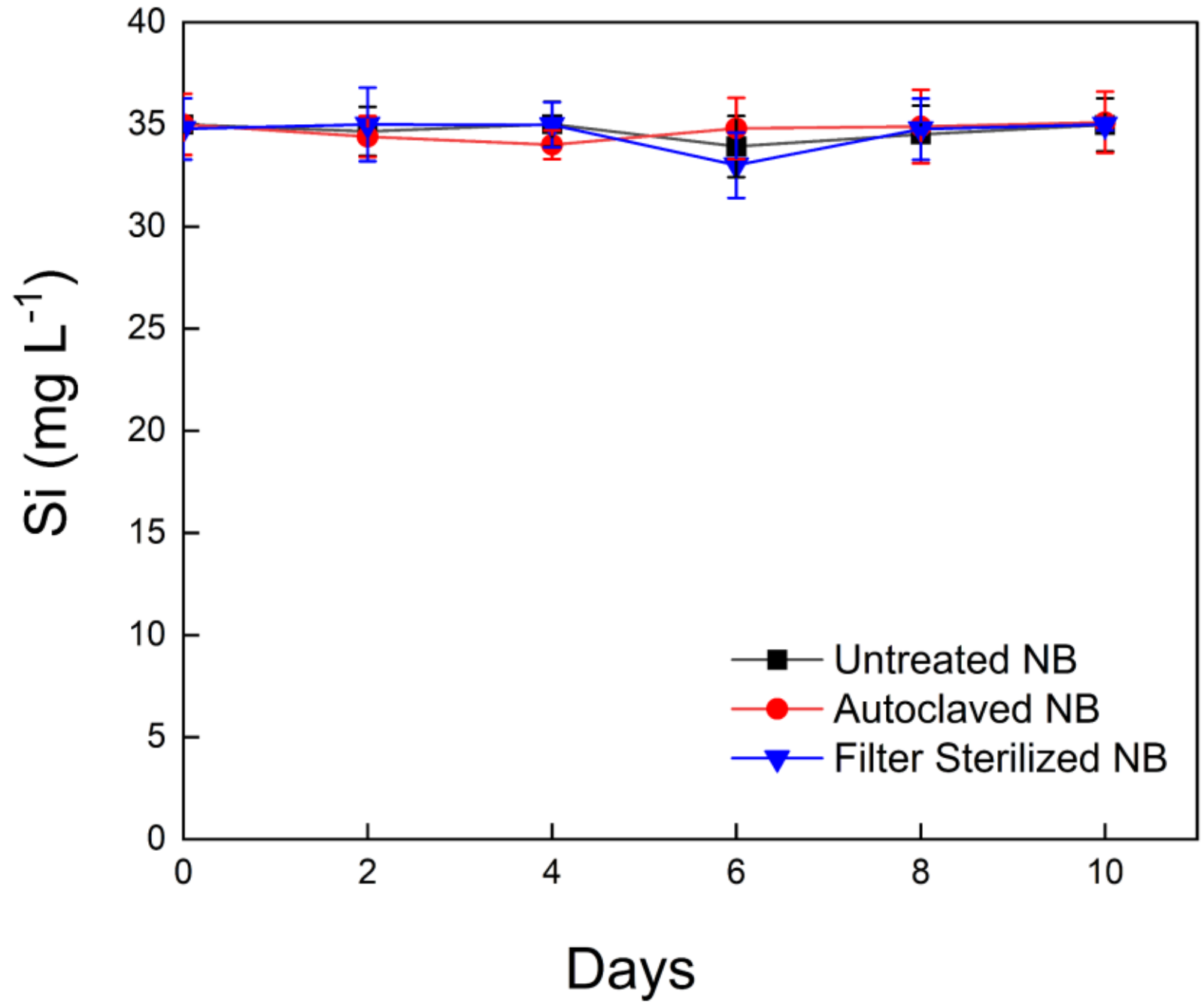

Fig S2. Si concentrations in natural brine without *Halamphora* innoculation. Untreated natural brine (black squares), autoclaved natural brine (red circles) and filter sterilized natural brine (Blue inverted triangle). Plots show concentration of Si measured in the solution over time. Data are represented as mean ± SD (standard deviation), n = 3 biological replicates.

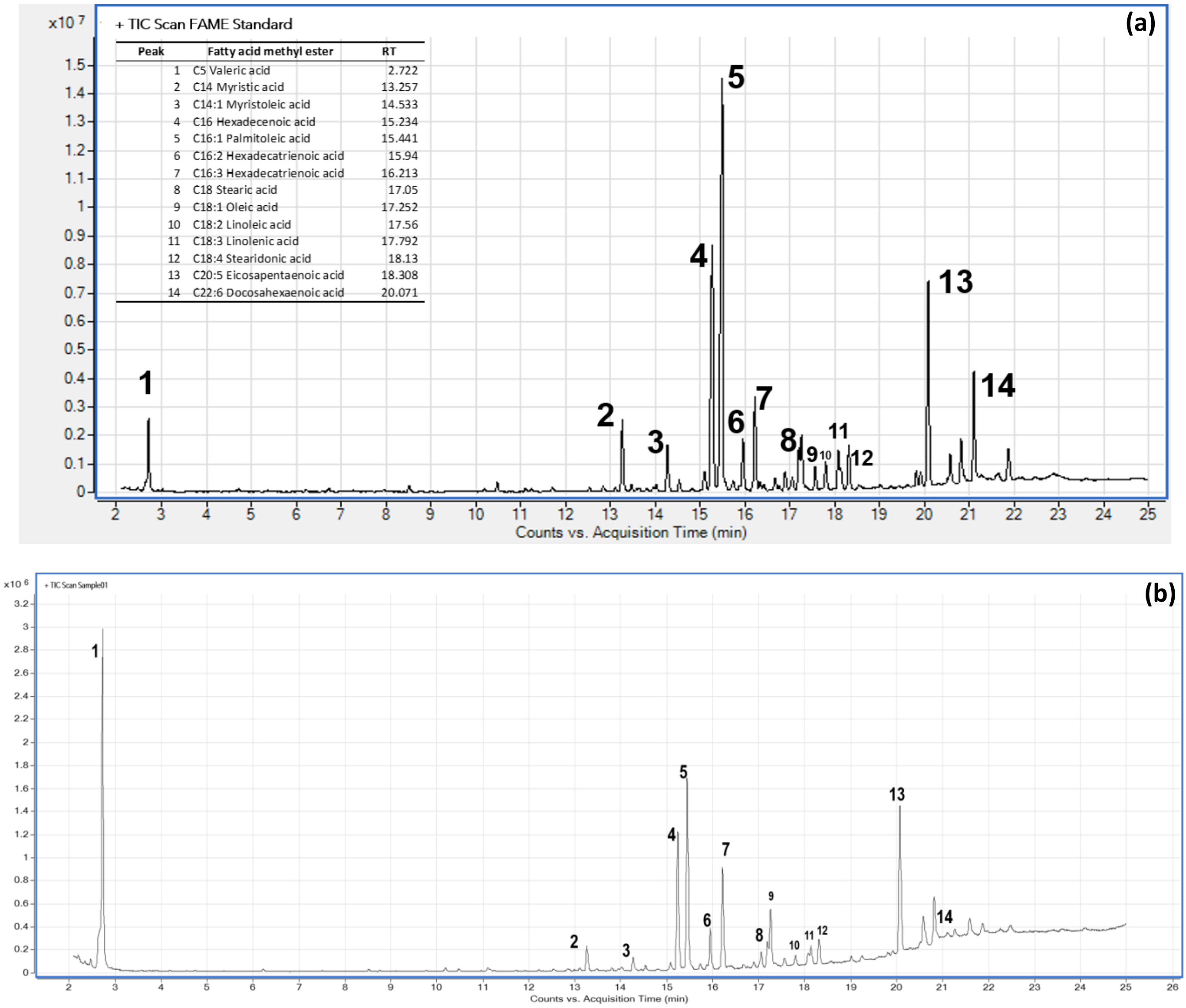

Fig S3. GC-MS Chromatograms showing separation of Fatty Acid Methyl Esters (FAMEs). (**a**) Total Ion Chromatogram (TIC) scan of a reference FAME standard mixture. Fourteen major peaks are numbered (1–14). The inset table lists the specific fatty acid methyl ester corresponding to each peak number and its observed retention time (RT) in minutes. (B) TIC scan of experimental sample, peaks are numbered to correspond with the identifications made using the standard mixture shown in panel A.

Table S1. Residual urea concentration in f/2 medium and natural brine, at different time points, data are represented as mean ± SD (standard deviation), n = 3 biological replicates.

	Urea concentration (mg L^{-1})	
**Time (in hours)**	**f/2 + urea**	**NB + urea**
0	26 ± 0.22	26 ± 0.44
12	20 ± 0.13	20.2 ± 0.32
24	14 ± 0.32	15 ± 0.2
36	8 ± 0.2	9 ± 0.12
Feed 1@ 36	26 ± 0.06	26 ± 0.06
48	18 ± 0.16	19 ± 0.16
60	11 ± 0.44	12 ± 0.44
72	4 ± 0.32	5 ± 0.32
Feed 2@ 72	26 ± 0.2	26 ± 0.2
84	17.2 ± 0.12	20.8 ± 0.12
96	11.2 ± 0.22	12.3 ± 0.22
108	4.3 ± 0.13	6.2 ± 0.13
Feed 3@ 108	26 ± 0.32	26 ± 0.32
120	11 ± 0.2	12 ± 0.2
132	5 ± 0.06	6 ± 0.06
144	0.5 ± 0.16	1 ± 0.44
Feed 4@ 144	26 ± 0.44	26 ± 0.32
156	9 ± 0.32	8 ± 0.2
180	1 ± 0.2	2 ± 0.12
Feed 5@ 180	26 ± 0.12	26 ± 0.06
192	12 ± 0.22	11 ± 0.16
204	5 ± 0.13	4 ± 0.44
216	0.5 ± 0.32	1 ± 0.32
Feed 6@ 216	26 ± 0.2	26 ± 0.12
228	14 ± 0.06	13 ± 0.22
240	8 ± 0.16	9 ± 0.13

Table S2. Operating conditions of scanning electron microscopy (SEM).

Operating conditions used for SEM analysis.	
**Accelerating voltage (kV)**	25
**Working distance (mm)**	20
**Electron beam current (μA)**	233
**Magnification (X)**	$5 - 10^5$
**Chamber pressure (Pa)**	10
**Interpretation software**	xT microscope control 6.2.7

Table S3. Operating conditions of Inductive coupled plasma optical emission spectroscopy (ICP-OES) and wavelengths at which the metals were detected.

Wavelengths and operating conditions used for ICP-OES determination of metals.	
**RF power (kW)**	1.2
**Plasma gas flow (L min^{-1})**	12
**Auxiliary Ar (L min^{-1})**	1.0
**Nebulizer Ar (L min^{-1})**	0.70
**Pump rate (mL min^{-1})**	1
**Readings/replicate**	3
**Wavelength (nm)**	Ca: 422.673; Si: 288.158; Mg: 285.213; Cu: 327.395; Fe: 238.204; Ni: 230.299; Mn: 294.921; Li: 670.783; Sr: 421.552; K: 769.897; B: 249.678; Na: 588.995; Sr: 421.552

Table S4. Operating conditions of Ion chromatography (IC) for anion detection in Natural brine.

Operating conditions used for IC determination of anions.	
**Eluent KOH (mM)**	30
**Operation mode**	Isocratic
**Flow rate (mL min $^{-1}$)**	0.38
**Suppressor (mA)**	29
**Column temperature (°C)**	30
**Pressure (psi)**	< 4000

Table S5. Silicon-Lithium separation factor ($SF_{\text{Si/Li}}$) for silica removal experiments in natural geothermal brine.

Note: Separation factor ($SF_{Si/Li}$) was calculated as the ratio of relative silica removal efficiency to relative lithium depletion. Because lithium retention exceeded $> 99\%$, measured changes in Li fall within the analytical precision limit (±1.5% RSD) of ICP-OES/MS. thus, SF values exceeding 100 demonstrate near-complete silica selectivity with negligible lithium uptake. To avoid reporting mathematical artifacts resulting from division by near-zero values, high-performing conditions are conservatively reported as $SF_{Si/Li} > 1{,}000$.

	Conditions	Separation factor ($SF_{Si/Li}$)
**Si removal from natural brine using different Diatom Species**	**NB (control)**	NA
	C. weissflogii	473
	F. pelliculosa	60
	Halamphora Sp.	>1000
	T. pseudonana	9
	**Swanpool isolate**	65
	**Kennall vale isolate**	78
**Effect of initial inoculum density of *Halamphora* sp. on Si removal from natural brine**	**NB_0 g/L**	NA
	**NB_0.05 g/L**	NA
	**NB_0.1 g/L**	266
	**NB_0.25 g/L**	966
	**NB_0.5 g/L**	>1000
	**NB_1.0 g/L**	>1000
**Effect of urea supplementati on on Si removal from natural brine**	**NB**	NA
	**NB + urea**	891
	**NB + urea feed**	>1000

Table S6. Comparative evaluation of silica-removal strategies for geothermal brine processing prior to Direct Lithium Extraction (DLE).

**Parameter / Technology**	**Silica seeds**	**Lime Precipitation**	**Metal Salt Coagulation**	**Acidification**	**Sorbent Adsorption**	**Bioprocess (Current study)**
**Silica Removal Efficiency**	57%	95.54%	85–95%	0% (Inhibition only)	80–90%	97%
**Lithium Retention in Brine**	80.55%	96.77%	88–95%	100%	92–97%	>99%
**Separation Factor ($SF_{Si/Li}$)**	0.94	1030.51	20–150	N/A	~100–300	3,851
**Chemical Reagent Inputs**	Silica gel, $(NH_4)_6Mo_7O_{24}$, HCL and NaOH	High ($Ca(OH)_2$, lime)	High (Fe^{3+}/ Al^{3+} salts)	High (H_2SO_4/HCl)	Medium (Sorbent replacement)	Low (0.2 kg urea / 1,000 L)
**Secondary Waste Generated**	solid silica particles	Hazardous chemical sludge	Toxic metal-silicate precipitate	None (Corrosive fluid)	Spent sorbent backwash	Zero hazardous sludge (Clean biomass)
**Co-Product Potential**	Recycling of Silica Seeds	None (Disposal cost)	None (Disposal cost)	None	None	High EPA+DHA
**Downstream DLE Impact**	High Na^+ and Cl^- ions into the solution can act as competing cations during downstream resin/sorbent-based DLE.	High pH requires acid correction	Metal cation contamination	Corrosive; fouls pH-sensitive resins	Minor	Optimal (Preserves neutral pH & Li^+)
**Primary Limitation**	The process depends on strict chemical dosing (HCl and NaOH) to initiate and control precipitation.	Higher-valent cations co-precipitate into the solid phase	Matrix contamination; disposal	Silica remains in brine; corrosion	Sorbent fouling; regeneration cost	Requires thermal cooling ($20-28°C$) & light
**Economic viability**	favourable with high reagent costs	Moderate with high waste handling costs	Unfavourable with toxic waste handling costs	Unfavourable	Moderate with high media replacement costs	Favourable with co-product sales
**References**	S1	S2	S3	S1	S4	This study

References

[S1] Setiawan, F.A., Rahayuningsih, E., Petrus, H.T.B.M., Nurpratama, M.I., and Perdana, I. (2019). Kinetics of silica precipitation in geothermal brine with seeds addition: minimizing silica scaling in a cold re-injection system. Geothermal Energy 7, 22. https://doi.org/10.1186/s40517-019-0138-3.

[S2] Spitzmüller, L., Goldberg, V., Held, S., Grimmer, J.C., Winter, D., Genovese, M., Koschikowski, J., and Kohl, T. (2021). Selective silica removal in geothermal fluids: Implications for applications for geothermal power plant operation and mineral extraction. Geothermics *95*, 102141. https://doi.org/10.1016/j.geothermics.2021.102141.

[S3] Gallup, D., Sugiaman, F., Capuno, V., and Manceau, A. (2003). Laboratory investigation of silica removal from geothermal brines to control silica scaling and produce usable silicates. Applied Geochemistry - Applied Geochemistry *18*, 1597–1612. https://doi.org/10.1016/S0883-2927(03)00077-5.

[S4] Yokoyama, T., Ueda, A., Kato, K., Mogi, K., and Matsuo, S. (2002). A Study of the Alumina–Silica Gel Adsorbent for the Removal of Silicic Acid from Geothermal Water: Increase in Adsorption Capacity of the Adsorbent due to Formation of Amorphous Aluminosilicate by Adsorption of Silicic Acid. Journal of Colloid and Interface Science *252*, 1–5. https://doi.org/10.1006/jcis.2002.8382.
